# Neonatal informatics: past, present and future

**DOI:** 10.1038/s41372-024-01924-4

**Published:** 2024-03-07

**Authors:** Shama Y. Patel, Jonathan P. Palma, Jeffrey M. Hoffman, Christoph U. Lehmann

**Affiliations:** 1 Division of Neonatology, Department of Pediatrics, The Ohio State University College of Medicine, Nationwide Children’s Hospital, Columbus, OH USA; 2 Division of Clinical Informatics, Department of Pediatrics, The Ohio State University College of Medicine, Nationwide Children’s Hospital, Columbus, OH USA; 3 Orlando Health Winnie Palmer Hospital, Orlando, FL USA; 4 Clinical Informatics Center, University of Texas Southwestern Medical Center, Dallas, TX USA

**Keywords:** Paediatrics, Health occupations

When computers and other technological advances entered the world of medicine and patient care, physicians marveled at the potential and immediately grasped the benefits of information that was instantly accessible. Today, we scroll through hundreds of MRI images in seconds, transmit prescriptions digitally, and review patients’ medical records on our smartphones and yet, compared to our expectations, digital innovations in medicine have still fallen short. The inefficiencies of the current electronic health record systems (EHRs) and the burden that resulted from implementing them are different than the advances that were envisioned and promised. In this article, we will describe the field of clinical informatics—a subspecialty that aims to address these gaps—discuss some of the historical context of neonatal informatics and present some recommendations to improve current documentation and EHR workflows within neonatology.

## A brief history of the digitization of health care

EHRs were implemented for use in patient care in the early 1960s followed shortly by development of automated clinical laboratory systems and automated multiphasic screenings using a programming language—MUMPS (Massachusetts General Hospital Utility Multi-Programming System)—that is still foundational in most commercial EHRs today [1–7]. Moving forward a few decades and EHR system adoption was rapidly accelerated through the Meaningful Use incentives of the Health Information Technology for Economic and Clinical Health (HITECH) Act in 2009 [8]. These incentives to adopt EHRs led to rapid technical implementation without the necessary sociotechnical tools namely trained informaticists to guide implementation within the context of local culture and allow for seamless integration of these new tools into clinical workflows [9].

The sudden ubiquitousness of these systems further underscored the need to have subspecialty-trained physicians with expertise in this area to help guide future technology implementations in clinical settings [10]. The groundwork for the establishment of the field of clinical informatics had been pioneered decades before and, in 2013, the first group of physicians became certified as sub-specialists in Clinical Informatics by the American Board of Preventive Medicine and the American Board of Pathology [11]. Since then, nearly 2800 physicians have become board certified in Clinical Informatics. These used their combined knowledge of clinical medicine and information technology to help implement and enhance all the various technologies that interface with healthcare today, from provider workflow optimization through remote patient monitoring programs and telehealth systems [12, 13].

## Neonatology on the forefront of digitization

Neonatologists were among the first pediatric sub-specialists to adopt electronic documentation and EHR systems long before the HITECH era. In 1992, Lowe, Ciszek and Gallaher described the implementation of NeoData (Isoprene Corp, Lisle IL), one of the first EHRs specifically designed for the Neonatal Intensive Care Unit (NICU). They discussed the importance of separation of the workflow for daily progress notes from other historical record-keeping and emphasized selectively moving only pertinent information forward automatically [14]. Fifteen years later, Drummond, in the first part of her 2009 article series entitled “Neonatal Informatics—Dream of a Paperless NICU,” described the shift from paper to stand-alone NICU-specific EHRs and found local, partial successes in her implementations [15].

## The central obstacles of EHR implementations

### Accelerated implementation

As a result of the rushed EHR implementations across all health care settings necessitated by the HITECH era there remains significant negative institutional memory within health care systems, as well as with individual physicians, about the increased burden of these new tools. There is also a perception that they are bloated and redundant record keeping systems that obstruct patient care [16]. When these systems are not designed efficiently there are known measurable negative impacts such as provider frustration, burnout and low satisfaction, in addition to that providers may be forced to develop workarounds and shortcuts that result in patient safety risks [17, 18].

### Digitization of paper based processes

A fundamental error in early neonatal EHRs, and a pervasive problem in all EHR implementations, is the goal to create digital analogs of existing paper workflows rather than understanding how new digital tools might enable novel and more efficient workflows that serve the same purpose [19]. As expected, we have now arrived at a consolidated EHR vendor market and have transitioned from free-standing, NICU-specific EHRs (e.g. NeoData, BabySteps [Pediatrix, Sunrise FL]) to commercial vendor products (e.g. EpicCare Inpatient [Epic System Corp, Verona WI], Cerner PowerChart [Oracle Corp, Austin TX]). Analogous to the transitions from three decades earlier, we are again struggling to make EHRs work for clinicians instead of clinicians working to satisfy the demands of the EHR [20].

### Managing burnout

EHR burden is often identified as a major cause of burnout due to the time spent in administrative tasks such as documentation [21], but local factors such as work culture may add to this perception [22]. The more effort spent sifting through EHR systems to locate important data or trends or performing mundane tasks, the less cognitive energy providers can dedicate to the care of patients [23]. A myriad hypotheses exist to explain the perceived deterioration of the patient-provider relationship [24], we postulate that the apparent intrusion into clinical care and workflow obstruction by EHR systems may be likely contributors.

A recurrent complaint about the EHR is that is forces the clinician into the role of a data entry technician. We assert that every member of the care team participates in rounds but their responsibilities and relationship to the patient are diverse and are be dictated by their role. This role division should translate to each individual’s interaction with the EHR.

## Redefining the role of progress notes

Historically in neonatology, the progress note served as an archive of a patient’s entire stay. It was the only place to safely communicate vital information between shifts and providers. In the transition from paper charting to early EHRs, the electronic progress note become our digital scratch pad, a necessary “external memory” to remind us of the last bilirubin, hematocrit, or cardiac echocardiogram for patients who had hospital stays marked in months not days.

As modern EHRs offer easier ways for information to be retained and accessed, the need for the progress note to house this information has become obsolete. While many neonatologists continue to identify external forces for inefficient documentation practices such as hospital administration, coders and payors, the reality is that much of the pain may be self-inflicted. The progress note length in the United States is four times that of other developed countries, and while some of this may be attributed to unique regulatory burdens in the United States, a sizeable portion stems from the culture of using daily documentation as a hand-off tool or running discharge summary [25].

## A way forward

How do we evolve EHR systems into valuable and trusted patient care tools? Firstly, clinicians must reclaim control over progress notes and restore them to their fundamental function as a clinical communication tool that captures the patient’s current clinical status and the decision-making for that day [26, 27].

Three major changes are needed to declutter the daily progress note and subsequently reduce daily documentation burden.

### Establish a hospital course

All historical patient information should be migrated into a separate area in the electronic chart – the hospital course. While structured differently in its content than the progress note, the hospital course is equally vital. It is as a living, breathing historical record of the patient that can be quickly accessed as needed.

### Create a NICU specific patient review screen

Development of a succinct patient review screen optimized for daily rounds and the information needed to make patient care decisions [19]. This area allows for review and retrieval of information such as vital sign summaries, laboratory results, ventilator settings, radiology reports, and similar data, making it unnecessary to include these details in the daily progress note or spend time hunting through the EMR for the most pertinent information.

### Use a standardized progress note template

Use of a standard note that pre-populates basic patient information and remains unburdened by granular details and allows the narrative of the patient to re-emerge [28]. In this new paradigm, every datum has a place where it logically lives in the chart and where critical information is retained without having to burden either the provider writing the note or those reading it with such overwhelming content that the progress note no longer serves its original purpose [19].

## A different type of continuing medical education

Just as providers keep up with continuously evolving procedural and other specialty specific knowledge, specialty-specific EHR training with appropriate clinical context is also vital. Training of a clinician is expensive, as we require an immense amount to do our jobs well including how to perform procedures, how to talk to families, how to analyze patient results, how to deliver bad news, how to educate trainees, and more. Paradoxically, we spend the most time each day with the EHR, for which we receive the least amount of training and guidance.

Documentation workflow practices are akin to procedural guidance; while minor nuances may exist in the way each of us approaches intubation, the ultimate result should be the same. Similarly, documentation workflows should be driven by agreed upon standards, best practices, and common templates rather than being dominated by individual providers’ stylistic preferences. Just as decreased variation in care improves patient care quality and outcomes, similar benefits exist in aligning clinical workflows and electronic documentation tools [29]. Regardless of the use of problem-based or systems-based charting, consensus and standardization are important. While this level of training may be costly, Stevens, Pantaleoni and Longhurst found a possible affordable solution by recruiting medical students as EHR trainers [30]. With the importance of physician satisfaction and the role the EHR has in efficiency and burnout, we must make significant changes to the way we document and train providers in the efficient use of EHRs [31].

## Actionable steps

1. Separate historical record-keeping from the daily progress note into a designated Hospital Course
2. Create dashboards that allow review of important daily patient data.
3. Build progress note templates that contain only clinically relevant information and assure pre-population of data. Review with billing and compliance for large gaps.
4. Understand that consistent, specialty-specific education is key to success of any technology implementations
5. Identify physician champions with expertise in clinical informatics who will be advocates for the clinicians with the IT team.

## Unique considerations of EHRs as research tools

A unique consideration in neonatology when modifying clinical documentation workflows is preservation of data reporting. While we cannot ignore data collection needs, they should not supersede workflow satisfaction and efficiency. The future of technology remains promising with the advent of artificial intelligence, machine learning, and large language models, such as GPT-4. The power of these technologies can only be fully realized when there is accepted best practice for documentation that generates standardized content that can be consumed, interpreted, and acted on by a number of AI tools. We must assure that the data collected in our EHR systems are timely, accurate, and complete [32, 33]. While our notes remain cluttered, bloated and without definition there is little utility to any application of higher level automated processing.

The neonatology workflow outlined here is attainable given the current state of EHR technology. Now we just need to add the proper support and training to best leverage the tools already available to us [34–36].
